# Optimal selection of resampling methods for imbalanced data with high complexity

**DOI:** 10.1371/journal.pone.0288540

**Published:** 2023-07-27

**Authors:** Annie Kim, Inkyung Jung

**Affiliations:** 1 Business Insight Team, Hyundai Autoever Corporation, Seoul, Republic of Korea; 2 Department of Biomedical Systems Informatics, Division of Biostatistics, Yonsei University College of Medicine, Seoul, Republic of Korea; Jeonbuk: Jeonbuk National University, DEMOCRATIC PEOPLE’S REPUBLIC OF KOREA

## Abstract

Class imbalance is a major problem in classification, wherein the decision boundary is easily biased toward the majority class. A data-level solution (resampling) is one possible solution to this problem. However, several studies have shown that resampling methods can deteriorate the classification performance. This is because of the overgeneralization problem, which occurs when samples produced by the oversampling technique that should be represented in the minority class domain are introduced into the majority-class domain. This study shows that the overgeneralization problem is aggravated in complex data settings and introduces two alternate approaches to mitigate it. The first approach involves incorporating a filtering method into oversampling. The second approach is to apply undersampling. The main objective of this study is to provide guidance on selecting optimal resampling methods in imbalanced and complex datasets to improve classification performance. Simulation studies and real data analyses were performed to compare the resampling results in various scenarios with different complexities, imbalances, and sample sizes. In the case of noncomplex datasets, undersampling was found to be optimal. However, in the case of complex datasets, applying a filtering method to delete misallocated examples was optimal. In conclusion, this study can aid researchers in selecting the optimal method for resampling complex datasets.

## 1. Introduction

The class imbalance problem is defined as the classification of datasets with unequal class distributions [1]. Class imbalance problems occur frequently in many real-world tasks, such as fraud detection, spam detection, and cancer prediction. In these operations, non-events occur often, resulting in "not spam," "not cancer," or "not fraud" results. Conversely, fraud, spam, and cancer occur rarely. Even if these events happen less frequently, their importance is not diminished. Minority classes are generally more interesting in terms of their learning tasks. Therefore, it is important to classify these infrequently occurring events properly.

However, imbalanced datasets create challenges in several machine learning tasks. This is because most machine learning algorithms assume a roughly balanced class distribution [2]. In case of class imbalance, the decision boundary is easily biased toward the majority class. Therefore, classification performance degrades when the imbalance ratio between the majority and minority classes is large. Several methods have been proposed to address this issue. The solutions can be divided into two groups: data-level and algorithm-level [3]. The data-level solutions, also known as resampling methods, change the balance between classes by modifying the data distribution [4–6]. In contrast, algorithm-level solutions impose a bias on the minority class by changing the search technique of the algorithm [7–9].

The greatest advantage of resampling methods is that they are more versatile. A dataset needs to be preprocessed only once for using it with different classifiers; hence, the computation to prepare the data is required only once. Therefore, resampling methods can be used in conjunction with various classification algorithms. Because of this advantage, numerous researchers have focused on resampling, which has resulted in the development of various resampling methods.

Resampling methods can be classified as either undersampling or oversampling. Undersampling deletes instances from the majority classes to maintain a balance between classes [10]. This technique provides a compact and balanced training set that reduces the learning cost. However, some important information may be lost [11]. It also increases the variance of the classifier. However, oversampling balances the number of samples between classes by adding instance copies to the minority class or by generating synthetic data [12]. The most widely used oversampling method is the synthetic minority oversampling technique (SMOTE) [4]. It creates new artificial data representing minority classes in its own manner.

However, the problem with SMOTE is that even if it achieves a better distribution between classes, it can yield worse results owing to the overgeneralization problem. In the overgeneralization problem, samples produced through oversampling techniques, which should be included in the minority class domain, are introduced into the majority-class domain. This overgeneralization problem can be aggravated in complex data settings. Applying oversampling under complex data settings can result in the creation of unnecessary minority class samples that do not simplify the learning of the minority class. It can also make the boundaries between classes unclear, rendering the classifier unusable. Therefore, data complexity is a major issue that causes overgeneralization. This study aims to address the problem of overgeneralization in complex data settings.

To mitigate the problem of overgeneralization in complex datasets, several studies have incorporated filters in oversampling methods. SMOTE is often used in conjunction with undersampling methods to remove samples considered detrimental to classification. The two most well-known methods are SMOTE-TomekLinks (TL) and SMOTE-edited nearest neighbor (ENN) [13,14]. Some filters, such as fuzzy rough set theory-based and ensemble-based noise filters, have been incorporated to enhance SMOTE [15–17]. Additionally, applying undersampling in certain cases can be a better solution in complex data settings. According to the work done by Park and Jung, undersampling methods perform better than other resampling methods despite the loss of information caused by undersampling [18]. Noisy or borderline samples can be filtered out from existing datasets.

The main purpose of this study was to suggest optimal resampling methods for handling imbalances in complex datasets. For this purpose, we investigated the relationships between data complexity and various resampling methods. To achieve this, six oversampling, ten undersampling, and ten filtering methods were applied to various simulated and real data, considering sample size range, imbalance ratio, and data complexity.

The remainder of this paper is organized as follows. In Section 2, various resampling methods are introduced. In Section 3, the application of resampling methods to the aforementioned simulated and real-life datasets are described, and the obtained results are analyzed. Thereafter, optimal resampling methods according to dataset complexity are suggested in Section 4. Finally, the paper is concluded in Section 5.

## 2. Methods

In this section, all resampling methods used in this study are explained. First, the most widely used classic oversampling and undersampling methods are introduced. Thereafter, a recently proposed filtering-based oversampling method is introduced as one of the methods to solve the overgeneralization problem. The resampling methods are listed in Table 1.

**Table 1:** Resampling methods.

Oversampling	Undersampling	Filtering
Random Oversampling	Random Undersampling	SMOTE-TL
SMOTE	Near-miss	SMOTE-ENN
ADASYN	Tomek Link	DSRBF
Borderline SMOTE	CNN	TRIM-SMOTE
SVM SMOTE	ENN	SMOTE-RSB*
K-means SMOTE	RENN	NRSBoundary-SMOTE
	All KNN	NEATER
	OSS	SMOTE-IPF
	NCR	SMOTE-FRST-2T
	IHT	NRAS

Adaptive synthetic (ADASYN), support vector machine (SVM), condensed nearest neighbors (CNN), repeated ENN (RENN), k-nearest neighbors (KNN), one-sided selection (OSS), neighborhood cleaning rule (NCR), instance hardness threshold (IHT), dynamic SMOTE radial basis function (DSRBF), neighboring rough set boundary (NRSBoundary), filtering of oversampled data using noncooperative game theory (NEATER), and iterative-partitioning filter (IPF), noise reduction a priori synthetic (NRAS).

### 2.1 Oversampling methods

Oversampling balances the number of samples between classes by adding an instance copy of an underrepresented class or by generating artificial data. We introduce three oversampling methods in addition to the three modified versions.

Random oversampling is the naive strategy for generating new examples. It randomly samples currently available samples with replacements. Therefore, the numbers of samples in the majority and minority classes become balanced, and the majority class does not take over another class during training. However, repeated sampling can result in overfitting.

**Fig 1:**
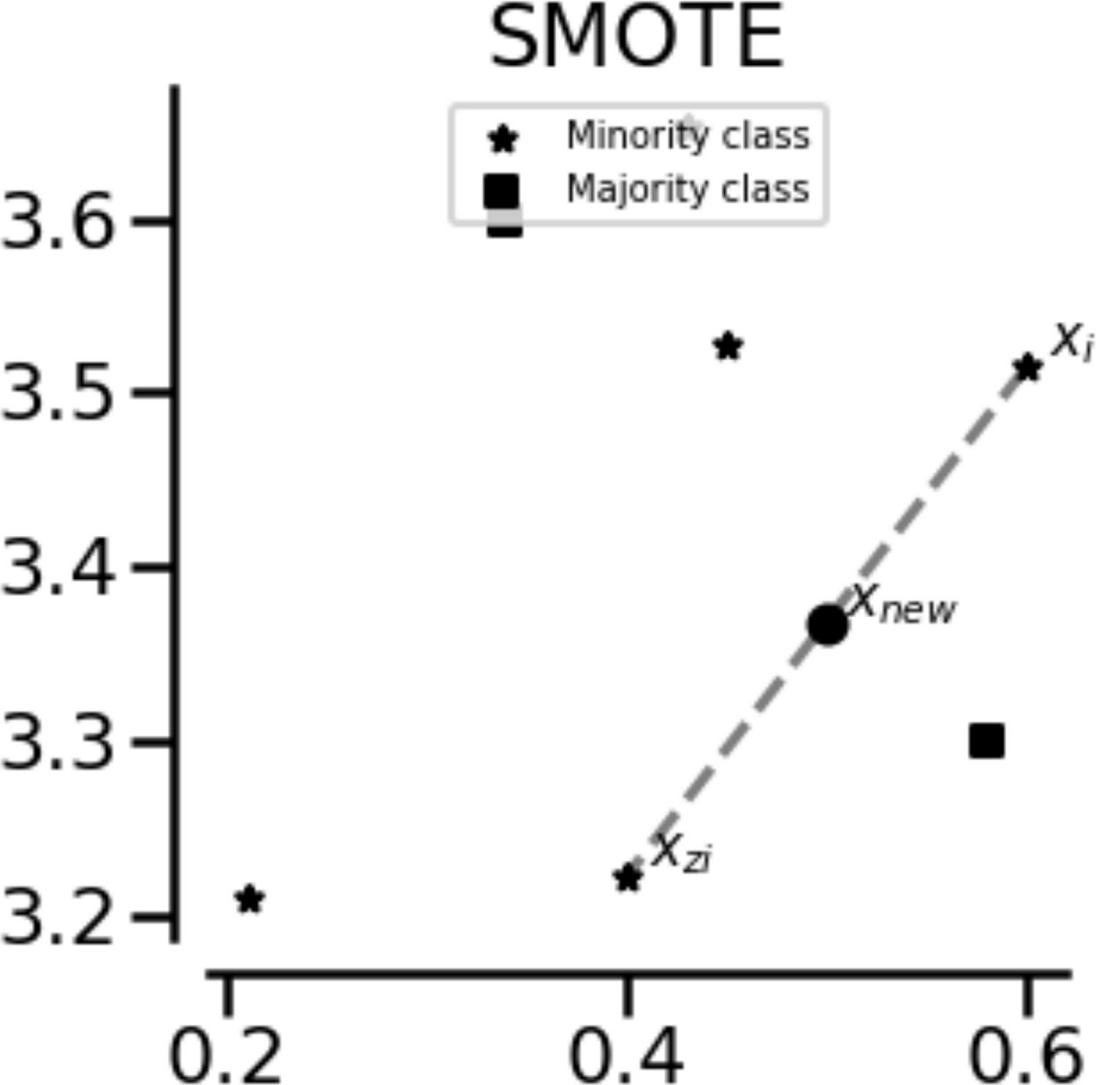
Visual representation of SMOTE. x_i_: Randomly selected minority class sample; x_zi_: Instance close to x_i_; x_new_: New artificial example generated by interpolation between two instances.

SMOTE is a popular method for oversampling a minority class. First, it randomly selects a minority class instance as the basis for generating new synthetic data. Thereafter, the closest neighbors of the same class are selected. Finally, random interpolation is performed between the two data points to obtain a new minority class instance [4]. SMOTE is illustrated in Fig 1.

ADASYN automatically determines the number of samples to be oversampled for each minority class by considering the dataset distribution [19]. A sample that needs to be oversampled is determined based on the learning difficulty. The learning difficulty can be quantified through the ratio of values belonging to the majority class to the KNN of values belonging to the minority class.

The oversampling methods introduced blindly generate the minority class sample without considering the majority class. This is particularly problematic for highly complex datasets and several methods have been developed to address this problem.

Borderline SMOTE determines the best candidates for oversampling in the entire dataset prior to oversampling [20]. This algorithm oversamples the samples that are close to the decision boundary, which is based on the premise that samples that are far from the boundary may contribute little to classification success.

SVM SMOTE and k-means SMOTE are variants of borderline SMOTE. SVM SMOTE uses SVM algorithms to detect the decision boundaries [21]. K-means SMOTE employs k-means clustering before applying SMOTE [22]. It groups samples together and creates new samples based on the clustering results.

### 2.2 Undersampling methods

Oversampling can result in overgeneralization. To address this problem, researchers have suggested applying undersampling methods instead. Undersampling is an efficient technique that does not add new data and reduces the risk of creating false decision boundaries generated by artificial samples.

There are two types of undersampling. The controlled undersampling technique allows a user-specified undersampling strategy to determine the number of samples to be sampled. However, the cleaning undersampling technique does not allow for this. Two controlled undersampling methods and eight cleaning undersampling methods are introduced in this section.

Random undersampling involves randomly selecting a sample from the majority class, with or without replacement. This is the simplest strategy for an imbalanced classification problem, similar to random oversampling. However, if the imbalance is severe, information loss may occur. Therefore, the performance may vary based on the degree of imbalance.

Unlike random undersampling, near-miss adds some heuristic rules. There are three versions of the near-miss algorithm. NearMiss-1 selects the majority-class sample with the smallest mean distance from the minority class to the nearest N sample. NearMiss-2 selects the majority-class sample with the smallest average distance from the minority class to the furthest N sample. NearMiss-3 is a two-stage algorithm that combines NearMiss-1 and NearMiss-2 [23]. This study used NearMiss-3 owing to its robustness to noise.

The methods described thus far allow users to set the number of samples to be undersampled. However, the following eight cleaning undersampling techniques do not allow for this. Therefore, after resampling, the number of minority- and majority-class samples can be different.

**Fig 2:**
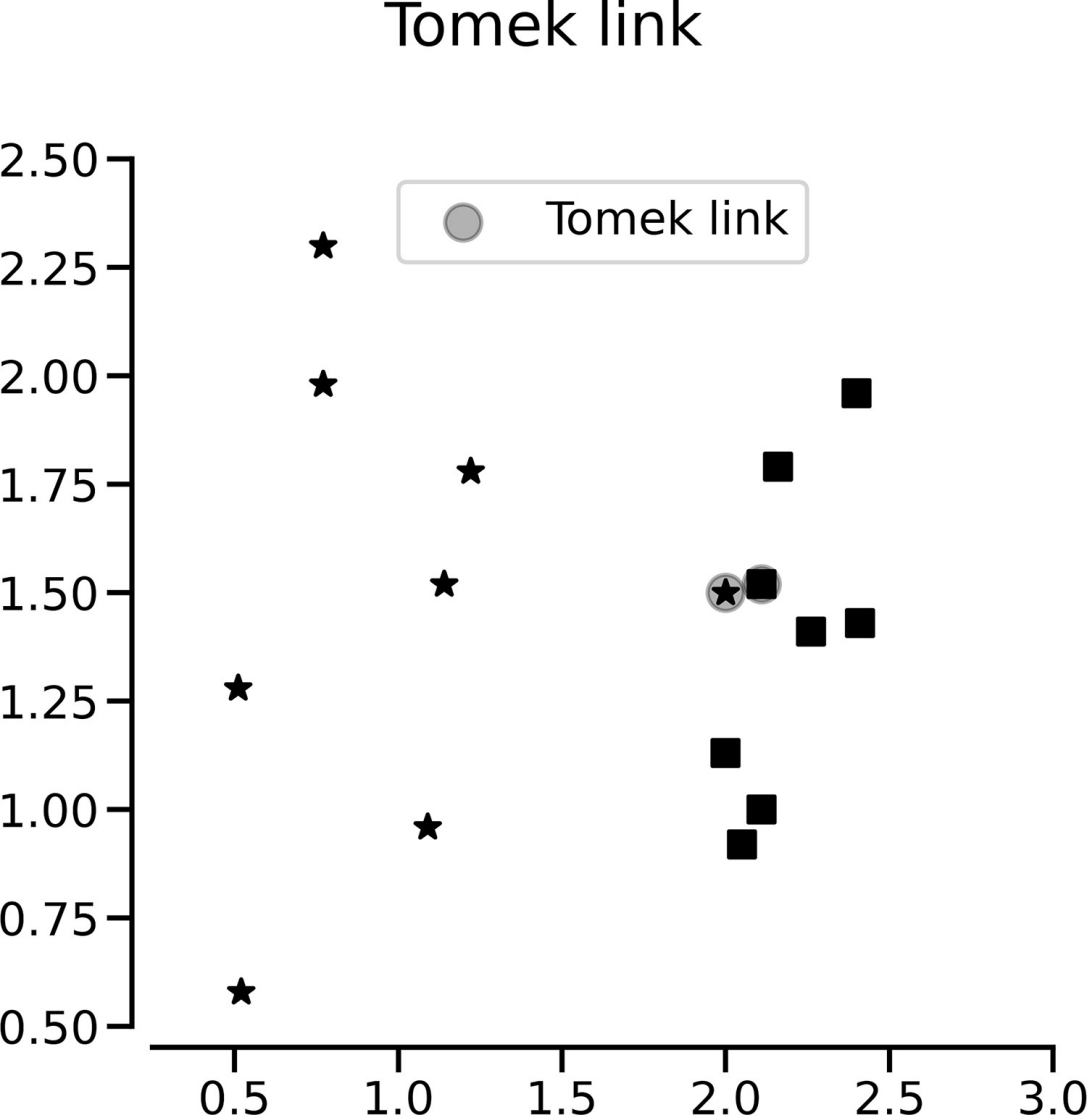
Illustration of TL.

The TL method performs undersampling by detecting TLs in the majority-class samples and removing them. TL is illustrated in Fig 2. For example, let d(.) be the distance between the two samples. If x and y belong to different classes and there is no sample z that satisfies the conditions d(x,y) < d(x,z) or d(x,y) < d(y,z), then the x, y pair is a TL [24]. Values classified as TLs are considered noise or borderline samples.

Using the 1-nearest neighbor rule, CNN iteratively determines whether the sample should be removed [25]. The aim of CNN is to find the smallest subset representing the majority class. However, CNN is known to be noise-sensitive.

ENN edits the dataset by removing samples that do not match their neighbors [26]. This algorithm is an extension of CNN and uses 3-nearest neighbors instead of one. RENN added few new rules to the original ENN [27]. It extends the ENN by repeating the algorithm multiple times. ALL KNN extends RENN by increasing the number of nearest neighbors at each iteration. OSS extends ENN by applying TLs and the 1-nearest neighbor rule to remove noisy samples [28]. NCR extends ENN by focusing on cleaning the dataset rather than condensing it [29]. If a value is classified as a majority class using 3-nearest neighbors, it is deleted. However, if the value is classified as a minority class, the majority-class samples in the 3-nearest neighbors are deleted.

IHT uses a trained classifier algorithm to remove samples with low predictive probabilities [30]. Unlike other distance-based classification methods, this algorithm applies a predictive probability based on the classification model.

### 2.3 Filtering methods

Another proposed solution to deal with the overgeneralization problem is adding filters to the oversampling methods. The overgeneralization problem can be resolved by cleaning the space resulting from oversampling. In this section, 10 filtering methods are introduced.

SMOTE-TL and SMOTE-ENN are the most typical filtering methods. The introduction of these methods were motivated by SMOTE’s well-known drawback of generating noisy examples. Each method adds TLs or ENNs after applying SMOTE to obtain a cleaner space [13,14].

DSRBF incorporates SMOTE with a memetic algorithm that optimizes radial basis function neural networks (RBFNNs), a clustering process, and a local search procedure [31].

TRIM-SMOTE uses TRIM as a preprocessing method [32]. It recursively splits an entire dataset into smaller clusters and searches for a precise minority region. The minority region obtained by TRIM is then used to generate new synthetic data.

SMOTE-RSB* introduced a new resampling method for highly imbalanced datasets [15]. After generating new synthetic examples using SMOTE, a selection method based on rough set theory is used to improve the quality. This process involves removing the generated examples that are not associated with the lower approximation of the minority class.

NRSBoundary-SMOTE is an extension of SMOTE-RSB* [33]. It divides the dataset into three groups. Of these three groups, it only oversamples the minority class samples in the boundary region, which indicates class overlapping.

NEATER does not consider the generated artificial data as minority class samples [34]. Rather, it keeps the generated samples unlabeled and determines the most likely class using a noncooperative game strategy. All generated artificial data that do not belong to the minority class are removed.

SMOTE-IPF removes noisy examples using an iterative ensemble-based noise filter [16]. A sample is removed if it is misclassified by more than half of the classifier.

SMOTE-FRST-2T employs the fuzzy rough set theory to remove data that do not belong to the majority-class region [17]. It then uses a double threshold to eliminate the original majority class and synthetic samples.

NARS improves the prediction of underrepresented samples containing noise by removing outliers [35]. It uses Bayes’ theorem to calculate the probability of a group membership. This includes minority class samples that do not appear as noise.

## 3. Evaluation of resampling methods on simulated data

### 3.1 Simulation framework

The generation method used was inspired by the work of Japkowicz and Stephen, who designed a similar framework to test the effect of resampling in complex settings [36]. However, their work only performed five generations for each simulated domain; therefore, their results cannot be considered reliable. Moreover, the performance metrics used by them had single threshold values, which may vary based on the selected threshold. Therefore, this study aimed to obtain more precise results by using better performance metrics. The simulated data for this study were generated as follows.

Six independent variables were selected, which comprised three continuous and three categorical variables, and a correlation of 0.3 was established between the them as follows:

$$\left[\begin{array}{c} X_{1}\\ X_{2}\\ X_{3}\\ \tilde{x_{4}}\\ \tilde{x_{5}}\\ \tilde{x_{6}} \end{array}\right]∼MVN\left([\begin{array}{c} 0\\ 0\\ 0\\ 0\\ 0\\ 0 \end{array}],[\begin{array}{cccccc} 1 & \rho & \rho & \rho & \rho & \rho \\ \rho & 1 & \rho & \rho & \rho & \rho \\ \rho & \rho & 1 & \rho & \rho & \rho \\ \rho & \rho & \rho & 1 & \rho & \rho \\ \rho & \rho & \rho & \rho & 1 & \rho \\ \rho & \rho & \rho & \rho & \rho & 1 \end{array}]\right),where\;\rho =0.3$$ (1)

$$X_{4}=\left\{ \begin{array}{c} 0\;if\;\tilde{x_{4}}<\Phi^{-1}(0.3)\\ 1\;o.w \end{array}\right.$$ (2)

$$X_{5}=\left\{ \begin{array}{c} 0\;if\;\tilde{x_{5}}<\Phi^{-1}(0.2)\\ 1\;o.w \end{array}\right.$$ (3)

$$X_{6}=\left\{ \begin{array}{c} 0\;if\;\tilde{x_{6}}<\Phi^{-1}(0.15)\\ 1\;o.w \end{array}\right.$$ (4)

Thereafter, the sigmoid function was applied to the generated data to make 0 < η > 1:

η=11+exp(−1*(1.1X1+0.9X2+0.7X3+X4+X5−X6+ϵ))ϵ∼N(0,1) (5)

**Fig 3:**
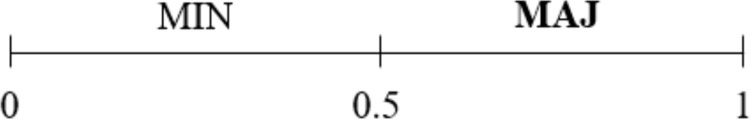
Backbone model under low complexity (c = 1). MIN: Minority class; MAJ: Majority class.

**Fig 4:**
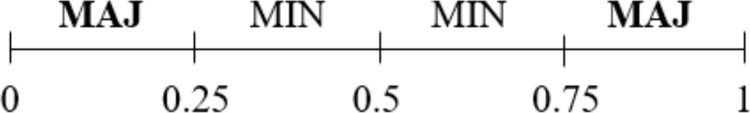
Backbone model under medium complexity (c = 2).

**Fig 5:**
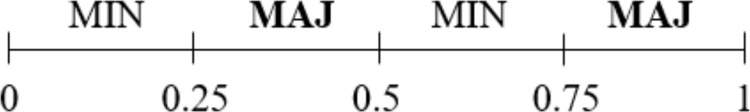
Backbone model under extreme complexity (c =2, but classes are spaced apart).

To control the complexity, imbalance, and sample size in binary problems, the backbone models illustrated in Figs 3–5 were applied to η. Three different levels of complexity were used, where level c corresponds to a backbone model composed of 2^c^ regular intervals. For example, the domain generated at c = 1 divides η by 0.5. Furthermore, when c = 2, η is divided into four intervals.

Finally, Eqs (6) and (7) were used to control the sample size and imbalance ratio. For three levels of sample size, imbalance ratio, and complexity (c = 1,2; s = 1,3,5; i = 1,3,5), the sample size of each interval is:

$$sample\;size\;of\;minority\;class\;interval=\left((\frac{5000}{32})*2^{s}\right)/2^{c}$$ (6)

$$sample\;size\;of\;majority\;class\;interval=\frac{((\frac{5000}{32})*2^{s})/2^{c}}{32/2^{i}}$$ (7)

By controlling the complexity (small, medium, or extreme), imbalance (i = 1, 3, 5), and size (s = 1, 3, 5), 27 domains were generated. Each domain was generated 50 times; the simulation settings are presented in Table 2.

**Table 2:** Simulation settings.

c	s	i	N	N+	IR
1, 2	1	1	166	156	15.6
		3	195	156	4
	3	1	664	625	16
		3	681	625	4
	5	1	2656	2500	16
		3	3125	2500	4

N: Number of samples; d: Number of variables; N+: Number of majority samples; IR: Imbalance ratio.

### 3.2 Other setup details

As most resampling methods showed similar performances when they were applied to various classification algorithms, this study only used the decision tree (DT) classifier as the classification method for the simulations. The test sets were created independently based on the complexity and imbalance ratio of each domain, and the size of the testing sets was set to 5000. The parameters for the DT classifier were selected through 3-fold cross validation.

The area under the precision-recall curve (AUPRC) was used for evaluation. In prior research, the selection of a suitable performance evaluation method has often been underestimated. However, selecting inappropriate metrics can result in misleading results because the metrics can vary based on the threshold selected [37]. Thus, using threshold-free metrics, such as area under the receiver operating characteristics (AUROC) or AUPRC, can be an effective approach.

**Fig 6:**
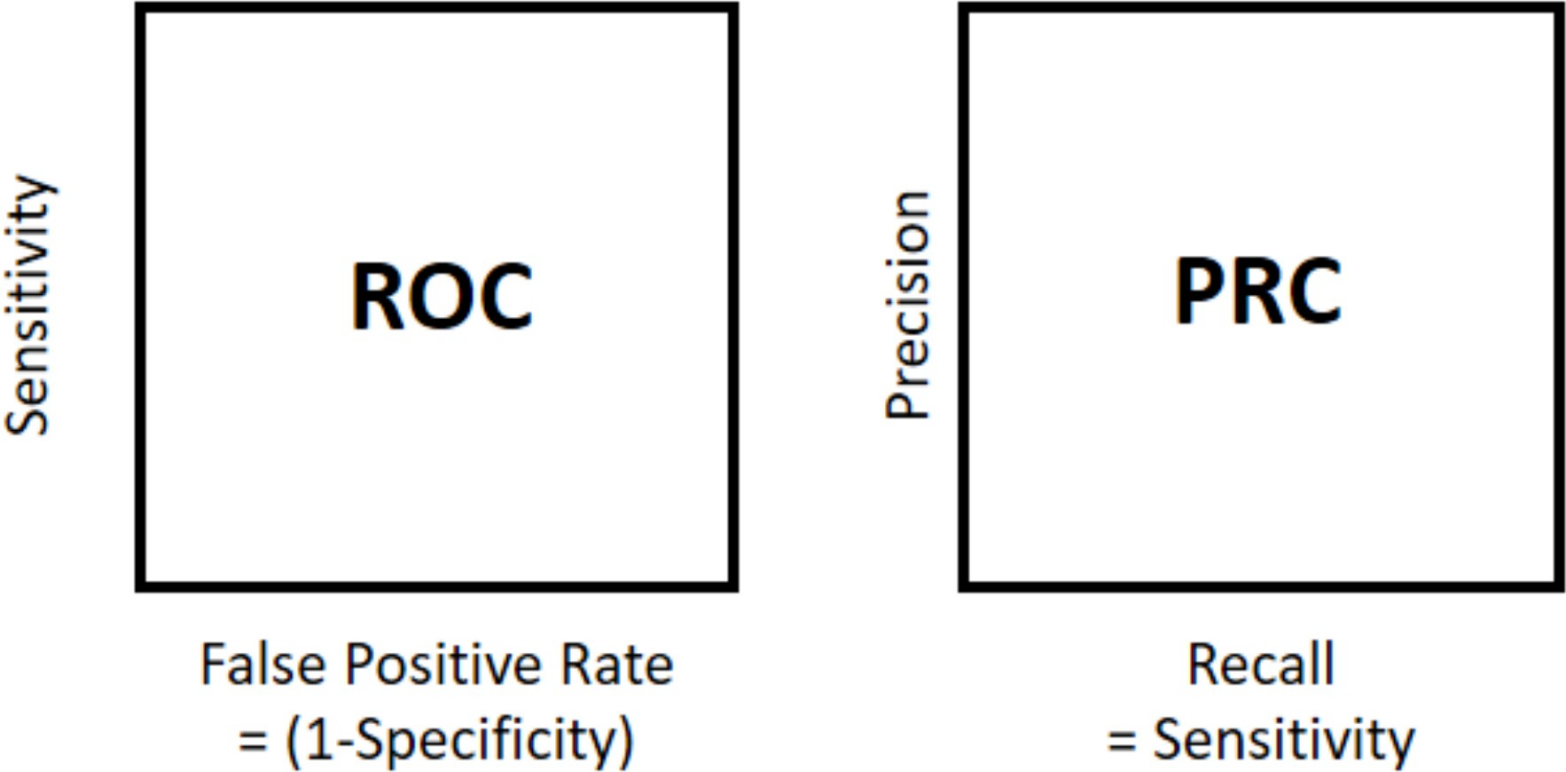
Differences between ROC and PR curves.

In AUROC, false- and true-positive rates are used for the x and y-axis, respectively. However, using AUROC can present an overly optimistic view of the classification performance if there is a large class imbalance [38]. Therefore, in unbalanced settings, using AUPRC helps in avoiding misinterpretation. In AUPRC, recall (sensitivity) and precision are used for the x and y-axis, respectively. It can reveal differences that are not visible in the ROC. The differences between the ROC curve of AUROC and PR curve of AUPRC are shown in Fig 6.

## 4. Results

**Fig 7:**
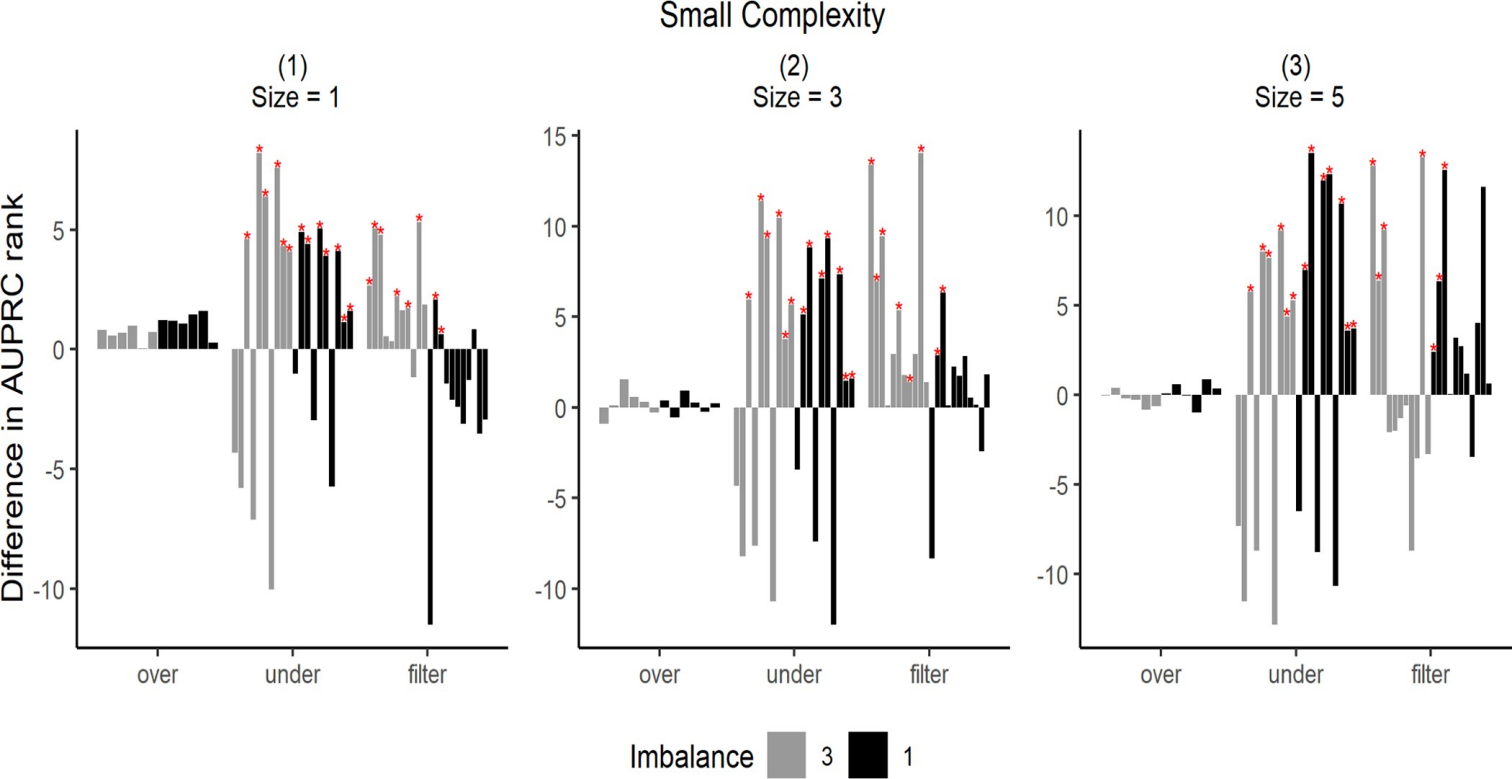
Differences in AUPRC ranks under low complexity. The star markers at the top of bars indicate significant performance gains.

**Fig 8:**
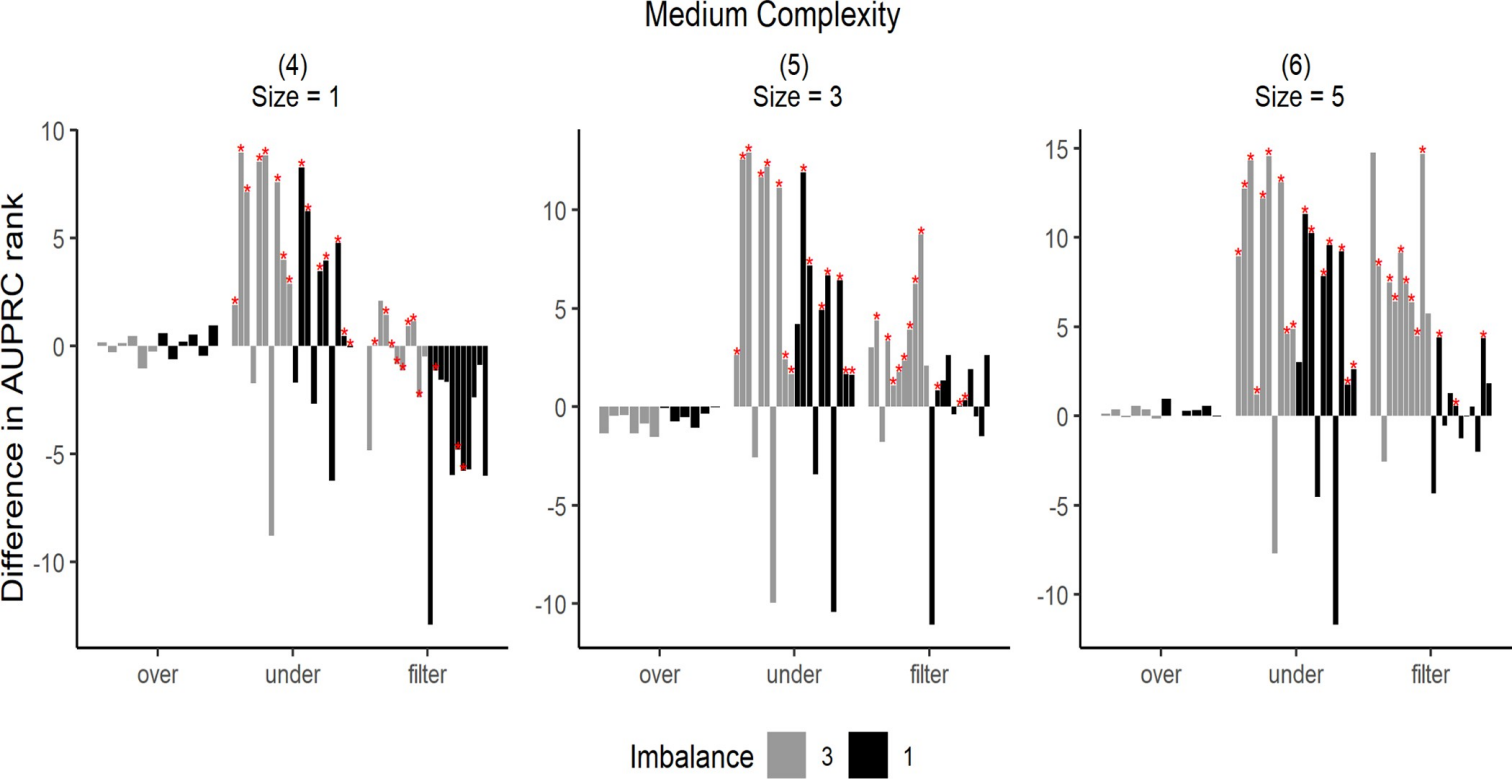
Differences in AUPRC ranks under medium complexity. The star markers at the top of bars indicate significant performance gains.

**Fig 9:**
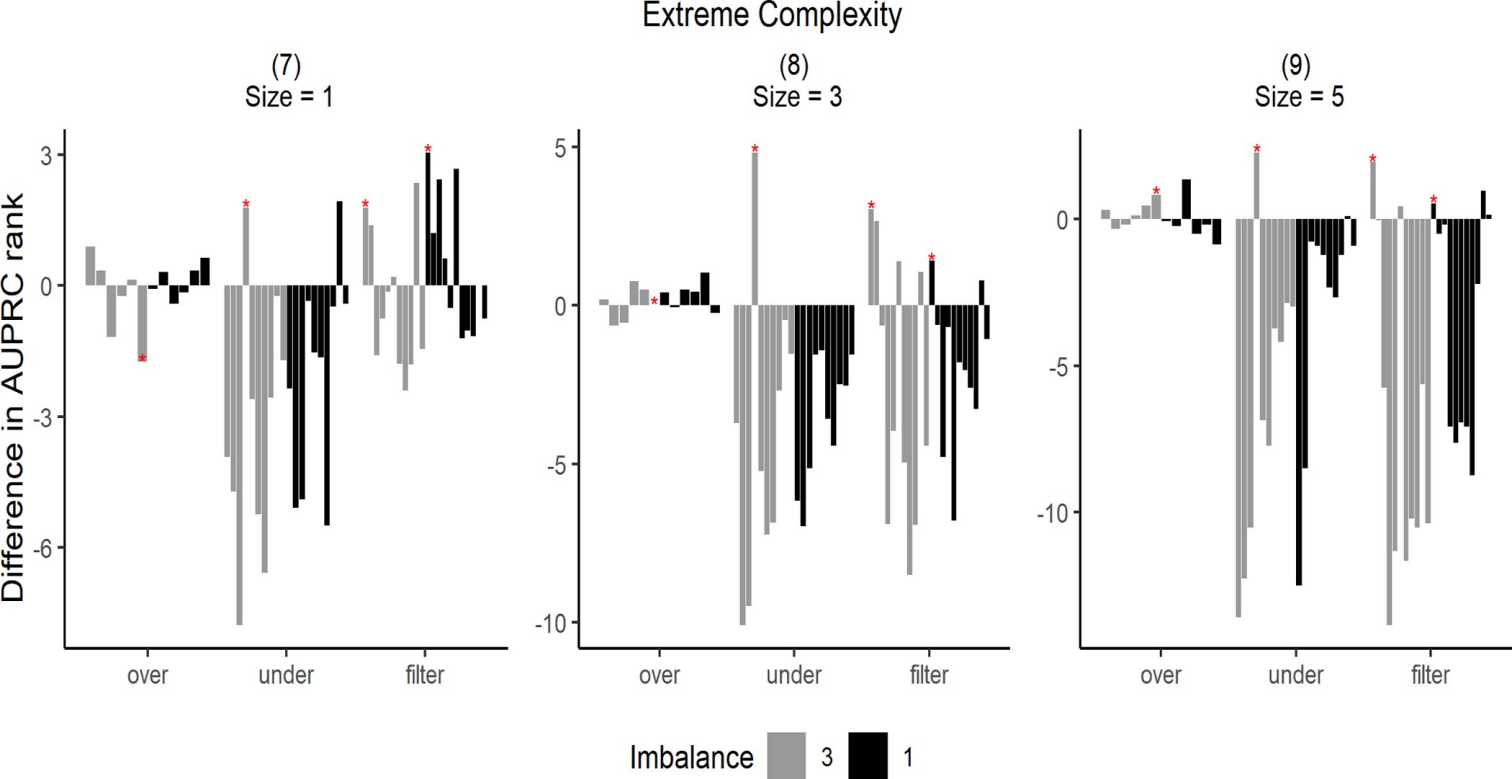
Difference in AUPRC ranks under extreme complexity. The star markers at the top of bars indicate significant performance gains.

The simulation results are shown in Figs 7–9. To illustrate the simulation results, the AUPRC value of each simulation data point was ranked from 1–26. The performance was then expressed by the difference in the ranking before and after resampling. Zero indicates an equal ranking before and after resampling, whereas positive/negative values indicate increase/decrease in ranking after resampling. Each bar in the graph represents the average difference in the ranking for the 50 simulation data.

Each graph in Figs 7–9 has different data complexity and size. Furthermore, in each graph, the x-axis represents each resampling method described in this study, and the y-axis represents the mean difference in the performance metrics. Colors indicate imbalance; light gray bars indicate lower imbalance than the dark ones. The star markers at the top of bars indicate significant performance gains based on paired t-tests.

It can be observed from Figs 7–9 that oversampling methods obtained lower ranks in all cases, regardless of the data complexity and imbalance. The performance was expected to decrease via oversampling as complexity increased owing to overgeneralization. However, the simulation results show that these problems occur even when the data are relatively less complex. Therefore, we concluded that creating artificial data to balance classes does not significantly increase performance under all complexities. However, compared with other resampling methods, oversampling showed more robust results without rapid rank changes, even under extreme complexity.

When data complexity was low or medium, undersampling methods obtained the highest ranks in most cases, and in the case of extreme data complexity, filtering methods obtained the highest ranks. Thus, it can be inferred that in the case of extreme data complexity, information loss because of undersampling has a considerable effect.

Furthermore, the undersampling results showed differences in ranks based on imbalance ratio. In cases of severe imbalance, the rankings were lower than those in less severe cases. This was because more majority-class samples were available for removal under sever imbalance. However, from the graphs (3), (6), and (9) shown in Figs 7–9, it can be observed that large sample sizes resulted in lower differences in ranking owing to the imbalance.

## 5. Evaluation of resampling methods on real data

In this section, the performances of six oversampling, ten undersampling, and ten filtering methods using four classification algorithms are compared. In this process, "complexity metrics" were used to represent the data characteristics. Through these metrics, the characteristics of resampling methods according to various data complexities were determined, and the optimal combination of classifiers and resampling methods was determined.

### 5.1 Data description

For the experiment, 109 labeled datasets were collected from the University of California Irvine (UCI) machine learning repository [39]. Most of them were based on multiclass datasets that were reorganized into binary problems by selecting some classes to compose the minority class and considering all other classes as the majority class. Some datasets were artificially generated, and most of them comprised real-life problems. The characteristics of the datasets are provided in Supporting Information (S1 Table).

Before applying the resampling methods, some feature encoding steps were performed to make the datasets applicable to the resampling methods and classification algorithms. Categorical values that contained less than five categories were one-hot encoded. For the others, the original values were retained to maintain a lower number of variables.

### 5.2 Complexity measures

The R package extended complexity library (ECoL) provides measures to characterize the complexity of classification problems based on overlapping features, neighborhood, and class imbalance.

Measuring complexity based on feature overlapping characterizes how informative the available features are for separating the classes. If there was one highly discriminative feature, the problem could be considered simpler than those with no such feature. The neighborhood-based complexity analyzes the neighborhoods of the data points and attempts to capture class overlap and the decision boundary shape. The complexity measure, based on imbalance, captures the differences in the number of samples per class in the dataset. We used three complexity measures, described in Table 3. Each measure ranges from 0 to 1, with higher values indicating higher complexity.

**Table 3:** Summary of complexity measures.

	Metric	Description
Overlapping	F3	Maximum individual feature efficiency. The ratio between the number of samples that are not in the overlapping region of two classes and the total number of samples.
Neighborhood	N2	Ratio of intra/extra class nearest neighbor distance. Computes the ratio of two sums: intra- and inter-class.
Imbalance	C2	Index computed for measuring class balance.

### 5.3 Other setup details

The classification methods used in this study to find the best combination of classifiers and resampling methods were DT, KNN, linear SVM, random forest (RF), and neural network. The training and test sets were divided into a 7:3 ratio, and the optimal parameters for each classification algorithm were set using 3-fold cross validation. For the evaluation, the AUROC was used in the same way as in the simulation study.

### 5.4 Mean differences in performances after resampling

**Fig 10:**
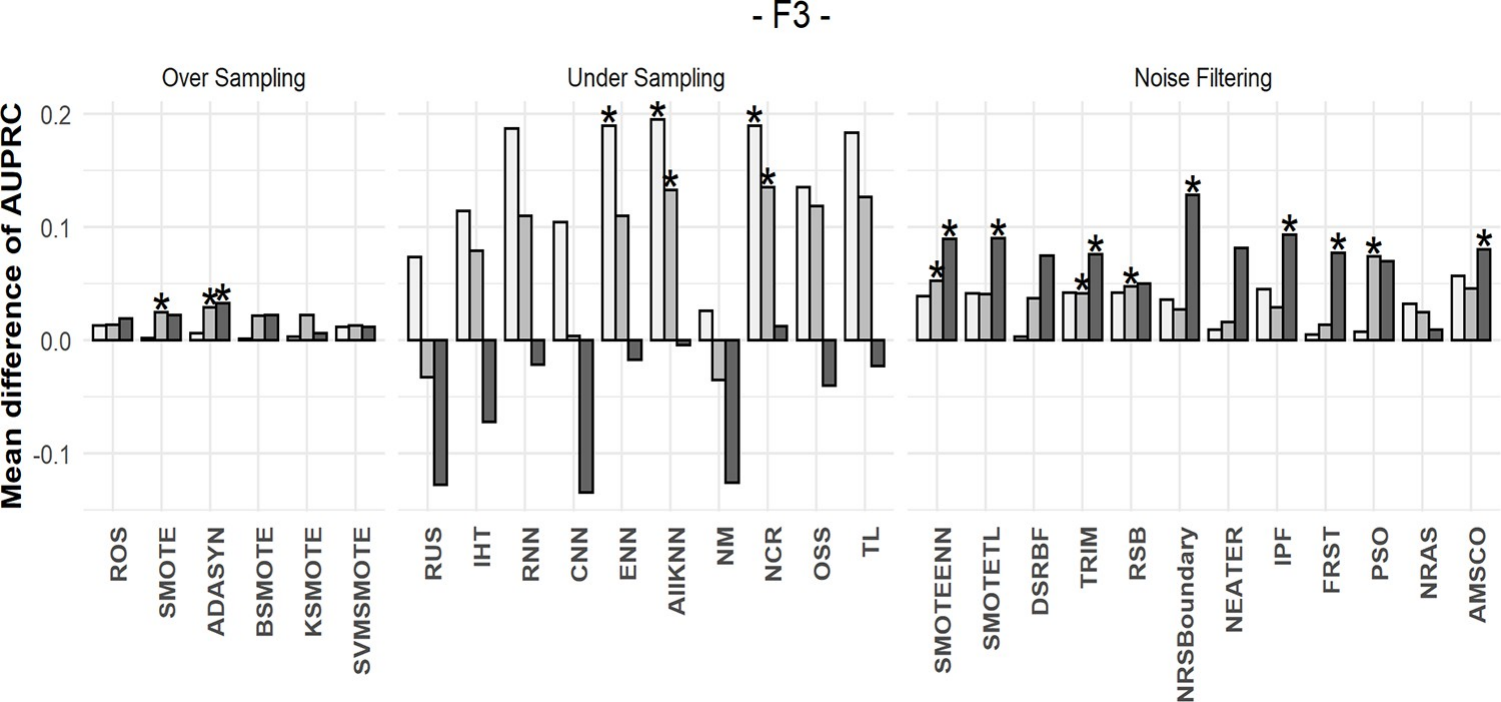
Mean differences in AUPRC values using F3. The more complex the dataset, the darker the color of the bar.

**Fig 11:**
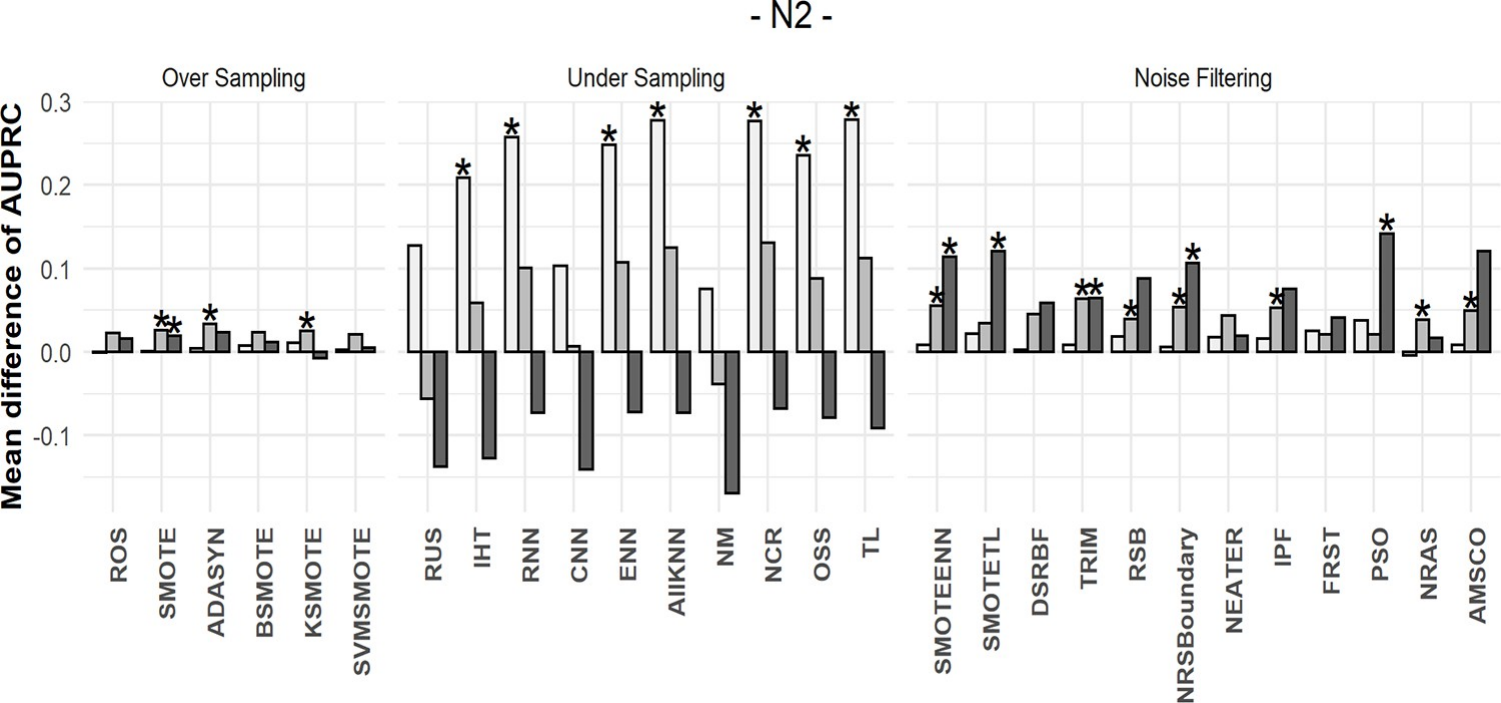
Mean differences in AUPRC values using N2. The more complex the dataset, the darker the color of the bar.

**Fig 12:**
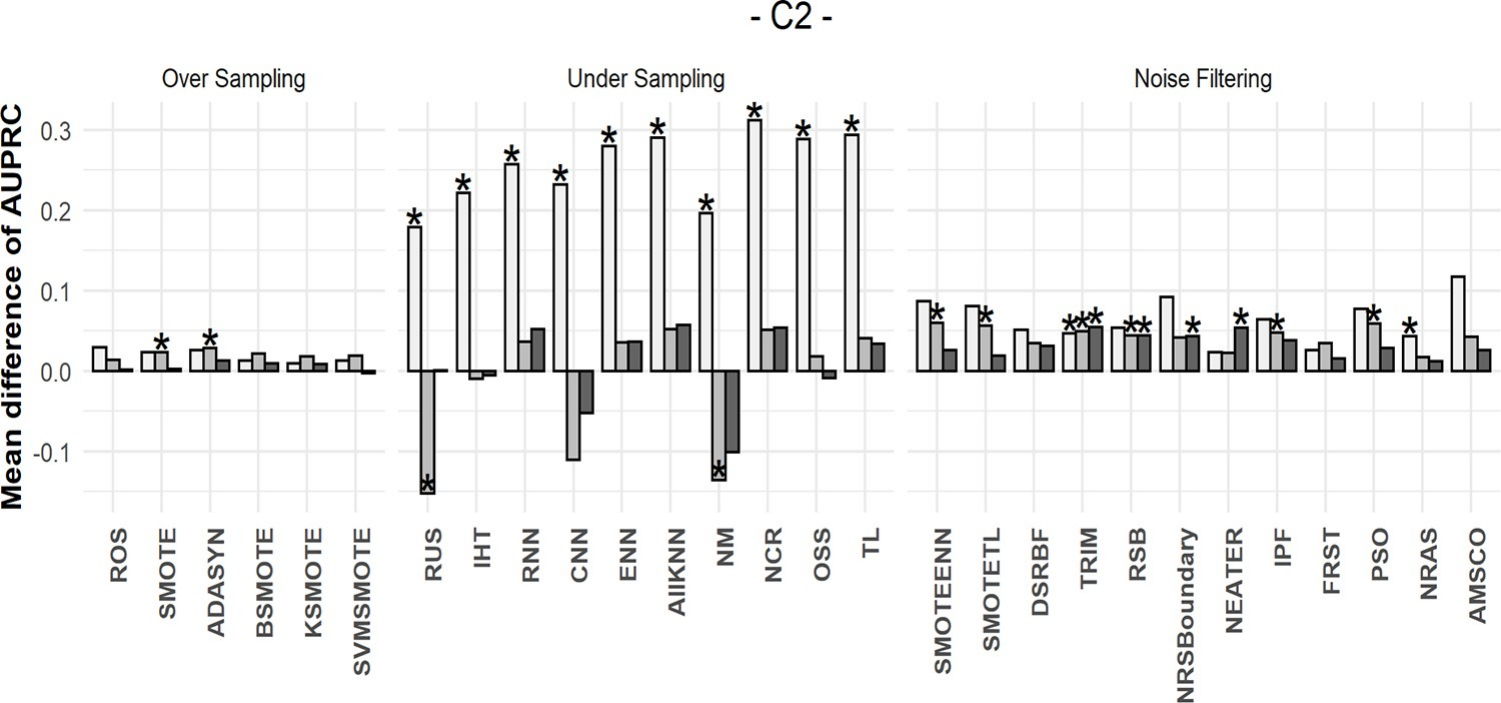
Mean differences in AUPRC values using C2. The more complex the dataset, the darker the color of the bar.

We calculated the AUPRC values before and after resampling for each dataset and then obtained the difference. For instance, the AUPRC value for the decision tree classifier was 0.309 for the original yeast3 dataset, whereas it was 0.027 after random undersampling. Thus, the AUPRC difference for the yeast3 dataset was -0.282. The mean differences between the original AUPRC values and those obtained after resampling for 109 datasets are shown in Figs 10–12. To determine the differences according to data complexity, each dataset was divided into three intervals using complexity measures: F3, N2, and C2. The more complex the dataset, the darker the color of the bar. A positive value indicates performance improvement, whereas a negative value indicates performance decrement.

Different methods were used to measure the complexity of the data; however, the data showed similar results. First, when oversampling methods were applied, there was little to no increase in AUPRC values for almost all complexities. This was consistent with the results of the simulation study.

In the undersampling results, when the complexity was low, most AUPRC values increased significantly. However, as complexity increased, the effect of undersampling became insignificant. Therefore, we concluded that information loss through undersampling was small for simple datasets. In such datasets, performance can be improved only by matching the balance of the two classes.

Conversely, the filtering method exhibits a different pattern. When the dataset was relatively simple, the mean increase in AUPRC values was similar to or less than that during undersampling. However, unlike undersampling, the filtering methods showed stable results, even for extremely complex datasets.

### 5.5 Results of the top 10 resampling and classifier combinations

In this section, the top 10 best-performing combinations obtained by applying all resampling methods and classifiers to the given data are discussed to determine the optimal combination for imbalanced and complex datasets. For example, in the yeast3 dataset, the best performing model was a combination of decision tree classifier and NEATER, which achieved an AUPRC of 0.721. The second and third ranked models were a combination of IPF+RF and a combination of CNN+RF, respectively, with AUPRCs of 0.701 and 0.696. To compare the results, the real data were divided into "complex" and "noncomplex" cases.

**Fig 13:**
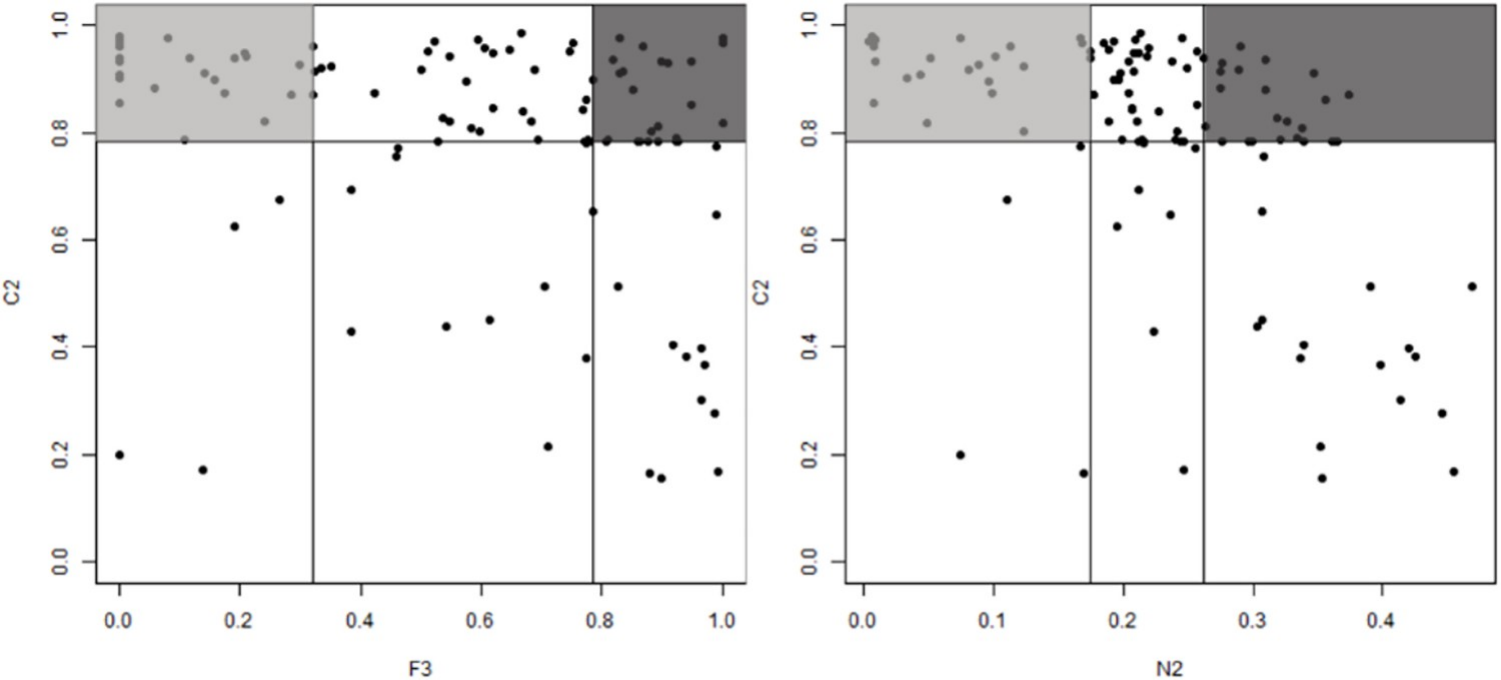
Complex and noncomplex areas in real datasets.

In this process, complexity measures were used to find complex and noncomplex cases among the given datasets. First, C2 was used to find the imbalanced data to which resampling should be applied. This study specified imbalanced data among all datasets as those that obtained C2 values in the top 25% or higher. Next, F3 and N2, which were well distributed among all complexity measures, were used to classify the complex and noncomplex cases. Data that obtained values in the top 25% were defined as complex cases, whereas those that obtained values in the bottom 25% were defined as noncomplex cases. In Fig 13, the parts colored in light gray indicate imbalanced and noncomplex areas, and those colored in dark gray indicate imbalanced and complex areas.

Tables 4 and 5 present the top 10 combinations of resampling methods and classifiers that obtained the highest average rankings based on AUPRC values. Table 4 lists the F3-based results for the noncomplex and complex cases. Table 5 lists the C2-based results for the noncomplex and complex cases.

**Table 4:** Top 10 combinations for complex and noncomplex datasets obtained using F3.

	Complex			Noncomplex		
	Resampling	Classifier	Average Rank	Resampling	Classifier	Average Rank
1	SMOTE-PSO	DT	35.92	SMOTE-IPF	RF	33.63
2	SMOTE	DT	38.08	SMOTE-FRST-2T	RF	34.90
3	NRAS	DT	39.10	NRSBoundary	RF	36.13
4	SMOTE-TL	RF	39.12	DSRBF	RF	40.04
5	SMOTE-IPF	RF	39.66	SMOTE-RSB*	RF	40.65
6	Random Oversampling	DT	40.16	NRAS	RF	41.42
7	ADASYN	DT	40.36	NCR	RF	42.94
8	SMOTE-TL	DT	40.52	SMOTE-PSO	RF	43.19
9	AMSCO	DT	42.44	SMOTE	RF	43.63
10	NRSBoundary	RF	42.56	Borderline SMOTE	RF	43.96

Light gray: Undersampling; dark gray: Oversampling; non-colored: Filtering. Of the 140 combinations, only the top 10 combinations are listed.

First, all classifiers that obtained high rankings were tree-based algorithms, such as RF and DT. Thus, it can be inferred that tree-based algorithms are effective for imbalanced datasets. Regarding resampling methods, filtering methods obtained the top rankings, regardless of the dataset complexity. The combinations of RF+SMOTE-IPF and RF+NRSBoundary ranked the highest in all cases.

**Table 5:** Top 10 combinations for complex and noncomplex datasets obtained using N2.

	Complex			Noncomplex		
	Resampling	Classifier	Average Rank	Resampling	Classifier	Average Rank
1	SMOTE-TL	RF	35.00	SMOTE-IPF	RF	30.13
2	SMOTE-IPF	RF	36.43	NRSBoundary	RF	35.69
3	SMOTE-RSB*	RF	37.00	DSRBF	RF	36.56
4	DSRBF	RF	38.64	ALL KNN	RF	39.83
5	SMOTE-FRST 2T	RF	41.00	SMOTE-FRST 2T	RF	40.33
6	NRSBoundary	RF	43.59	SMOTE-TL	RF	41.85
7	SMOTE-PSO	DT	45.16	SMOTE	RF	42.92
8	SMOTE-PSO	RF	46.84	SVM SMOTE	RF	43.13
9	NCR	RF	46.91	none	RF	43.94
10	SMOTE	DT	46.93	ALL KNN	DT	44.21

Light gray: Undersampling; Dark gray: Oversampling; non-colored: Filtering; none: No resampling. Of the 140 combinations, only the top 10 combinations are listed.

In complex cases, undersampling methods obtained relatively lower ranks, whereas filtering methods obtained higher ranks. This result was consistent with that of the simulation study. In Table 4, there is no undersampling method in the top 10 combinations, and in Table 5, there is one undersampling method that ranks ninth. Among the filtering methods, SMOTE-TL was suitable when F3 was high and SMOTE-PSO was suitable when N2 was high.

The ranks of undersampling methods were relatively high for noncomplex cases compared to complex cases. When N2 was low, the combinations of ALL KNN with RF and DT ranked high. However, even when the complexities were low, the filtering methods ranked high. Specifically, RF+SMOTE-FRST-2T and RF+DSRBF obtained high average ranks for noncomplex cases.

From these tables, we can observe that it is unusual for oversampling methods to rank high in both complex and noncomplex cases. However, oversampling methods with different features were selected based on their complexity. In the case of complex datasets, algorithms that do not control the region to be oversampled ranked high, whereas in the case of noncomplex datasets, those that control the region ranked high.

## 6. Discussion

The objective of this study was to provide an optimal resampling method for complex datasets. In several studies, oversampling methods have resulted in poor classification for imbalanced datasets. This study suggested two alternative approaches for overcoming this problem for complex datasets.

Through various simulation scenarios, we observed that applying oversampling methods resulted in poor overall performance. Therefore, we suggest that an undersampling or filtering method should be applied instead. In the case of noncomplex datasets, undersampling was found to be optimal. However, performance varied according to the degree of imbalance. In the case of complex datasets, applying a filtering method to delete misallocated examples was optimal.

Based on real data analysis, the best combinations of classifiers and resampling methods for each data characteristic were provided. Overall, the combination of RF and filtering methods obtained the best performance. SMOTE-IPF+RF and NRSBoundary+RF combinations obtained high rankings for all four cases. For complex and imbalanced datasets, in addition to the aforementioned filtering methods, SMOTE-TL and SMOTE-PSO obtained high rankings when they were combined with tree-based classifiers. For noncomplex and imbalanced datasets, the combination of ALL KNN, an undersampling technique, and a tree-based classifier obtained high performance.

Research on resampling methods has opened new opportunities to improve the classification performance for imbalanced datasets. Many researchers have contributed to the development of resampling methods owing to their convenience and versatile features. However, the empirical behavior of resampling methods is highly dependent on the characteristics of the observed data. We believe that this study can contribute to the improvement of classification performance for imbalanced and complex datasets and further the development of resampling methods.

## Supporting information

S1 Table Summary of 109 real data sets used in the analysis. (DOCX)

S1 File This file contains the R and Python codes generated for this study. (ZIP)
