# Supplementary material for: Optimal selection of resampling methods for imbalanced data with high complexity
Source: PLoS One. 2023 Jul 27;18(7):e0288540. doi: 10.1371/journal.pone.0288540 (PMC10374143; doi:10.1371/journal.pone.0288540)
Supplement: S1 Table — (DOCX) [file pone.0288540.s001.docx]

S1 Table. Summary of 109 real data sets used in the analysis

| Name | N | d | +N | IR |
| --- | --- | --- | --- | --- |
| Glass1 | 214 | 9 | 138 | 1.82 |
| Wisconsin | 683 | 9 | 444 | 1.86 |
| Pima | 768 | 8 | 500 | 1.87 |
| Ecoli 0 vs 1 | 219 | 7 | 143 | 1.88 |
| Iris0 | 150 | 4 | 100 | 2.00 |
| Glass0 | 214 | 9 | 144 | 2.06 |
| German | 1000 | 29 | 700 | 2.33 |
| Yeast1 | 1484 | 10 | 1055 | 2.46 |
| Haberman | 305 | 3 | 225 | 2.81 |
| Vehicle1 | 845 | 18 | 627 | 2.88 |
| Vehicle3 | 846 | 18 | 629 | 2.90 |
| Vehicle2 | 846 | 18 | 634 | 2.99 |
| ADA | 4147 | 47 | 3118 | 3.03 |
| Glass 0-1-2-3 vs 4-5-6 | 214 | 9 | 163 | 3.20 |
| Vehicle0 | 846 | 18 | 647 | 3.25 |
| Ecoli1 | 335 | 7 | 258 | 3.35 |
| Hepatitis | 155 | 19 | 123 | 3.84 |
| SPECT_F | 267 | 44 | 212 | 3.85 |
| New thyroid1 | 215 | 5 | 180 | 5.14 |
| KC1 | 2109 | 21 | 1783 | 5.47 |
| Ecoli2 | 335 | 7 | 284 | 5.57 |
| Segment0 | 2308 | 23 | 1979 | 6.02 |
| Glass6 | 214 | 9 | 185 | 6.38 |
| Yeast3 | 1484 | 10 | 1321 | 8.10 |
| Ecoli3 | 336 | 7 | 301 | 8.6 |
| Pageblocks0 | 5472 | 10 | 4913 | 8.79 |
| Ecoli 0-3-4 vs 5 | 200 | 7 | 180 | 9.00 |
| Yeast 2 vs 4 | 514 | 8 | 463 | 9.08 |
| Ecoli 0-6-7 vs 3-5 | 222 | 7 | 200 | 9.09 |
| Ecoli 0-2-3-4- vs 5 | 202 | 7 | 182 | 9.10 |
| Glass 0-1-5 vs 2 | 172 | 9 | 155 | 9.12 |
| Yeast 0-3-5-9 vs 7-8 | 506 | 10 | 456 | 9.12 |
| Yeast 0-2-5-6 vs 3-7-8-9 | 1004 | 10 | 905 | 9.14 |
| Yeast 0-2-5-7-9 vs 3-6-8 | 1004 | 10 | 905 | 9.14 |
| Ecoli 0-4-5 vs 5 | 203 | 6 | 183 | 9.15 |
| CM1 | 498 | 23 | 449 | 9.16 |
| Ecoli 0-1 vs 2-3-5 | 244 | 7 | 220 | 9.17 |
| Ecoli 0-2-6-7 vs 3-5 | 224 | 7 | 202 | 9.18 |

N, number of samples; d, number of variables; +N, number of majority samples; IR, imbalance ratio

Table (continued)

| Name | N | d | +N | IR |
| --- | --- | --- | --- | --- |
| Glass 0-4 vs 5 | 92 | 9 | 83 | 9.22 |
| SATIMAGE | 6435 | 7 | 185 | 9.25 |
| Ecoli 0-3-4-7 vs 5-6 | 257 | 7 | 232 | 9.28 |
| Yeast 0-5-6-7-9 vs 4 | 528 | 10 | 477 | 9.35 |
| Vowel 0 | 988 | 13 | 898 | 9.98 |
| Vowel 0 | 988 | 13 | 898 | 9.98 |
| Ecoli 0-6-7 vs 5 | 220 | 6 | 200 | 10.00 |
| Glass 0-1-6 vs 2 | 192 | 9 | 175 | 10.29 |
| Ecoli 0-1-4-7 vs 2-3-5-6 | 336 | 7 | 307 | 10.59 |
| Led7digit 0-2-4-6-7-8-9 | 443 | 7 | 406 | 10.97 |
| Ecoli 0-1 vs 5 | 240 | 6 | 220 | 11.00 |
| Glass 0-6 vs 5 | 108 | 9 | 99 | 11.00 |
| Glass 0-1-4-6 vs 2 | 205 | 9 | 188 | 11.06 |
| Glass2 | 214 | 9 | 197 | 11.59 |
| Ecoli 0-1-4-7 vs 5-6 | 332 | 6 | 307 | 12.28 |
| Cleveland 0 vs 4 | 177 | 23 | 164 | 12.62 |
| Ecoli 0-1-4-6 vs 5 | 280 | 6 | 260 | 13.00 |
| PC1 | 1109 | 21 | 1032 | 13.40 |
| Shuttle c0 vs c4 | 1829 | 9 | 1706 | 13.87 |
| Yeast 1 vs 7 | 459 | 7 | 429 | 14.30 |
| Sylva | 13086 | 212 | 12281 | 15.26 |
| Glass4 | 214 | 9 | 201 | 15.46 |
| Ecoli4 | 335 | 7 | 315 | 15.75 |
| Page blocks 1-3 vs 4 | 472 | 10 | 444 | 15.86 |
| Abalone9 19 | 731 | 8 | 689 | 16.40 |
| Dermatology6 | 358 | 129 | 338 | 16.90 |
| Zoo3 | 101 | 16 | 96 | 19.20 |
| Glass 0-1-6 vs 5 | 184 | 9 | 175 | 19.44 |
| Hypothyroid | 3163 | 25 | 3012 | 19.95 |
| Shuttle c2 vs v4 | 129 | 9 | 123 | 20.5 |
| Shuttle 6 vs 2-3 | 230 | 9 | 220 | 22.00 |
| Yeast 1-4-5-8 vs 7 | 693 | 10 | 663 | 22.10 |
| Glass5 | 214 | 9 | 205 | 22.78 |
| Yeast 2 vs 8 | 482 | 10 | 462 | 23.10 |
| Lymphography normal | 148 | 23 | 142 | 23.67 |
| Flare F | 1066 | 11 | 1023 | 23.79 |
| Car good | 1728 | 6 | 1659 | 24.04 |
| Car vgood | 1728 | 6 | 1663 | 25.58 |

N, number of samples; d, number of variables; +N, number of majority samples; IR, imbalance ratio

Table (continued)

| Name | N | d | +N | IR |
| --- | --- | --- | --- | --- |
| Kr vs K zero one vs draw | 2901 | 6 | 2796 | 26.63 |
| Kr vs K one vs fifteen | 2244 | 6 | 2166 | 27.77 |
| Yeast4 | 1484 | 10 | 1433 | 28.10 |
| Winequality red 4 | 1599 | 11 | 1546 | 29.17 |
| Poker 9 vs 7 | 244 | 25 | 236 | 29.50 |
| Kddcup guess | 1642 | 38 | 1589 | 29.98 |
| Yeast 1-2-8-9 vs 7 | 947 | 10 | 917 | 30.57 |
| Abalone 3 vs 11 | 502 | 8 | 487 | 32.47 |
| Windquality white 9 vs 4 | 168 | 11 | 163 | 32.60 |
| Yeast 5 | 1484 | 10 | 1440 | 32.73 |
| Kr vs K three vs eleven | 2935 | 6 | 2854 | 35.23 |
| Winequality red 8 vs 6 | 6556 | 11 | 638 | 35.44 |
| Ecoli 0-1-3-7 vs 2-6 | 281 | 7 | 274 | 39.14 |
| Abalone 17 vs 7-8-9-10 | 2338 | 8 | 2280 | 39.31 |
| Abalone 21 vs 8 | 581 | 8 | 567 | 40.50 |
| Yeast6 | 1484 | 10 | 1449 | 41.40 |
| Winequality white 3 vs 7 | 900 | 11 | 880 | 44.00 |
| Winequality red 8 vs 6-7 | 855 | 11 | 837 | 46.50 |
| Kddcup land vs portsweep | 1061 | 40 | 1040 | 49.52 |
| Abalone 19 vs 10-11-12-13 | 1622 | 8 | 1590 | 49.69 |
| Kr-vs-K zero vs eight | 1460 | 6 | 1433 | 53.07 |
| Winequality white | 1482 | 11 | 1457 | 58.28 |
| Poker 8-9 vs 6 | 1485 | 25 | 1460 | 58.40 |
| Shuttle 2 vs 5 | 3316 | 9 | 3267 | 66.67 |
| Winequality red 3 vs 5 | 691 | 11 | 681 | 68.10 |
| Abalone 20 vs 8-9-10 | 1916 | 8 | 1890 | 72.69 |
| Kddcup buffer | 2233 | 31 | 2203 | 73.43 |
| Kddcup land vs satan | 1610 | 30 | 1589 | 75.67 |
| Kr-vs-K zero vs fifteen | 2193 | 6 | 2166 | 80.22 |
| Poker 8-9 vs 5 | 2075 | 25 | 2050 | 82.00 |
| Poker 8 vs 6 | 1477 | 25 | 1460 | 85.88 |
| Kddcup rootkit | 2225 | 47 | 2203 | 100.14 |
| Abalone19 | 4174 | 9 | 4142 | 129.44 |

N, number of samples; d, number of variables; +N, number of majority samples; IR, imbalance ratio
